# Therapeutic effect of mesenchymal stem cells on histopathological, immunohistochemical, and molecular analysis in second-grade burn model

**DOI:** 10.1186/s13287-021-02365-y

**Published:** 2021-05-29

**Authors:** Doaa Ramadan I. Abdel-Gawad, Walaa A. Moselhy, Rasha Rashad Ahmed, Hessah Mohammed Al-Muzafar, Kamal Adel Amin, Maha Mohamed Amin, El-Shaymaa El-Nahass, Khaled Abbas Helmy Abdou

**Affiliations:** 1 Toxicology and Forensic Medicine Department, Faculty of Veterinary Medicine, Beni-Suef University, Beni-Suef, 62511 Egypt; 2 Zoology Department, Faculty of Science, Beni-Suef University, Beni-Suef, Egypt; 3 Department of Chemistry, College of Science, Basic & Applied Scientific Research Center, Imam Abdulrahman Bin Faisal University, P.O. Box 1982, Dammam City, 31441 Saudi Arabia; 4 Basic and Applied Scientific Research Center, Imam Abdulrahman Bin Faisal University, P.O. Box 1982, Dammam, 31441 Saudi Arabia; 5 Pathology Department, Faculty of Medicine, Mansoura University, Mansoura, Egypt; 6 Veterinary Pathology Department, Faculty of Veterinary Medicine, Beni-Suef University, Beni-Suef, Egypt

**Keywords:** BM-MSCs, Burn, Histopathological, Immunohistochemical, qPCR

## Abstract

**Background and aim:**

Deleterious cutaneous tissue damages could result from exposure to thermal trauma, which could be ameliorated structurally and functionally through therapy via the most multipotent progenitor bone marrow mesenchymal stem cells (BM-MSCs). This study aimed to induce burns and examine the effect of BM-MSCs during a short and long period of therapy.

**Material and methods:**

Ninety albino rats were divided into three groups: group I (control); group II (burn model), the animals were exposed to the preheated aluminum bar at 100*°*C for 15 s; and group III (the burned animals subcutaneously injected with BM-MSCs (2×10^6^ cells/ ml)); they were clinically observed and sacrificed at different short and long time intervals, and skin samples were collected for histopathological and immunohistochemical examination and analysis of different wound healing mediators via quantitative polymerase chain reaction (qPCR).

**Results:**

Subcutaneous injection of BM-MSCs resulted in the decrease of the wound contraction rate; the wound having a pinpoint appearance and regular arrangement of the epidermal layer with thin stratum corneum; decrease in the area percentages of ADAMs10 expression; significant downregulation of transforming growth factor-β (TGF-β), interleukin-6 (IL-6), tumor necrotic factor-α (TNF-α), metalloproteinase-9 (MMP-9), and microRNA-21; and marked upregulation of heat shock protein-90α (HSP-90α) especially in late stages.

**Conclusion:**

BM-MSCs exhibited a powerful healing property through regulating the mediators of wound healing and restoring the normal skin structures, reducing the scar formation and the wound size.

## Introduction

Injury of the skin or of any other tissues due to excessive heat or cold, chemicals, electricity, radiation, or friction is called a burn [1], which represents one of the greatest public health concerns, resulting in significant patient morbidity and mortality [2]. Annually, burns account for more than 7.1 million injuries, the loss of almost 18 million disability-adjusted life years (DALYs), and more than 250,000 deaths worldwide [3].

Concerning the affected layers of skin from the applied heat, the severity of burns is categorized into different degrees, ranging from first (most severe) to fourth degree (least severe) [4]. Patients are considered at risk of developing life-threatening metabolic disorders when burns of second to fourth degree cover more than 20% of the total body surface area and are regarded as incompatible with survival when the scale of burning reaches nearly 50% of the TBSA [5]. For accurate investigation of the burn pathophysiology, animal models have been established which provide beneficial information in helping to develop new therapies that reduce the severity of the burns [6]. Subsequently, numerous animal models have been used to determine the effects of the burn trauma and the efficacy of the local therapy and the administration of the systemic drug [7].

Burns could be treated through natural products [8], anti-inflammatory drugs such as traditional non-steroidal drugs [9] and nontraditional ones as opioids [10], excision and grafting [11], dermal analogs, and skin substitutes [12].

In severe cutaneous burn injury, the natural healing process and the endogenous source of SCs in the basal layer are limited in their usage for repairing the extensive and deep damage [13]. The most recent and emerging branch of medical science that deals with this condition of body repairing insufficiency is regenerative medicine [14]. SCs represent the front-line source of regenerative medicine for the regeneration of tissue and organ anomalies caused by congenital defects, diseases, and age-related effects [14]. The aim of the application of regenerative medicine in burn therapy involves the acceleration of re-epithelialization and reconstruction of the functional skin with hair follicles, sweat glands, and dermal capillaries which might be achieved by SC therapy [15].

Minimal risk of hypertrophic scarring [16], high-quality therapy for skin coverage, efficiency, and low morbidity rates are the advantages of using SCs in regenerative medicine in treating burn injury [17]. Moreover, SCs might identify other systemic effects of burn injury, including hypermetabolism and inflammation [18]. MSCs can restore the normal skin architecture and function after injury [19]. In this respect, they do not only accelerate the rate of wound closure, but they also enhance the quality of the wound healing and the function of the regenerated skin [20].

In light of this, the current study aims to determine the efficiency of BM-MSCS in treating the deep second-grade burn and determining the expression rate of healing mediators at acute and late stages of wound healing.

## Material and methods

### Chemicals

Dulbecco’s modified Eagle’s medium (DMEM), fetal bovine serum (FBS), penicillin-streptomycin solution, and trypsin/EDTA were obtained from Lonza, Belgium. Sodium hydrogen carbonate was purchased from LOBA Chemie, India. Culture consumables and culture flasks were purchased from Greiner Bio-One (Germany).

### Isolation of BM-MSCs

#### Preparation of the complete culture medium

It is composed of DMEM (89%) supplemented with FBS (15%), penicillin-streptomycin solution (1%), and sodium hydrogen carbonate (0.36%) [21].

#### BM-MSC isolation and culturing

BM-MSCs could be segregated feasibly because of their tendency to adhere to the plastic surface when they are maintained in standard culture conditions.

The procedures of isolation and culturing were done according to Chaudhary and Rath [22] with some modifications.

For isolation, 4–6-week-old male albino rats were sacrificed via decapitation, followed by complete sterilization of the whole-body surface through spraying ethyl alcohol (70%); then, the tibiae and femur bones were dissected out and the whole surrounding tissue removed. Under complete septic conditions in a Biobase vertical laminar flow cabinet (Biobase, model: BBS V1300; NO-51, South Gongye Road, Jinan, Shandong Province, China), the epiphysis of the previously dissected bones was cut off just below the growth plate by using sterile fine scissors, for the harvesting of the cells.

The bone marrow was flushed with DMEM, and the solution was collected in a Falcon tube (15 ml), centrifuged for 5 min at 3000 rpm for dislodgement and separation of the cells; the supernatant was discarded, followed by rapid washing of the cell pellet with phosphate-buffered saline (PBS), and suspended in the previously prepared complete culture medium. The viable and dead cells were counted post-staining with trypan blue solution (0.2%) which detects the viability of the cells, and counting was done via the hemocytometer at ×100 magnification (the number of the viable cells was relative to the total number of the cells).

In T-25 cm^2^ sterile Greiner cell culture flasks with canted neck, 2.5× 10^6^ cells were seeded at a density of 1 × 10^6^ cells/cm^2^ area and then incubated in a 5% CO_2_-humidified incubator (Biobase, Model: BJPX-C50; South Gongye Road, Jinan, Shandong Province, China) at 37°C. Floating and non-adherent cells, as well as dead cells, were removed after 4 days from incubation, and through 7–10 days of culturing and incubation, the adherent cells were washed with sterile PBS twice (pre-warmed at 37°C) and trypsinized with 1–2 ml of trypsin (0.25%)/EDTA (1 mM) (pre-warmed for 2–3 min at 37°C).

The detachment of the adherent cells was warranted via examining the cells under an inverted biological microscope (Novel, model: NIB-100; Jiangsu, China). Adding 3–5 ml complete culture medium for stopping the action of trypsin, which was followed by the collection of cells and centrifugation for 5 min at 3000 rpm, the cells were re-suspended in the culture medium after washing two times in an incomplete DMEM. After that, the cells were counted, and viability was assessed by adding an equal volume of trypan blue (0.2%).

### Animals

Ninety healthy male albino rats (*Rattus norvegicus*) weighing about 110–150 g were used. Animals were maintained under observation for about 7 days, for ensuring the absence of any intercurrent infections. In the animal house department, they were kept in plastic cages with stainless steel cover, at normal temperature, with enough food and water ad libitum. All animal procedures were approved via the Animal Ethics Committee of Zoology Department, Faculty of Science, Beni-Suef University.

### Experimental design

The experimental design includes 3 groups: group I (6 rats for the control negative group) were not exposed to burn. Group II (42 rats for the burn group) animals were pre-anesthetized with atropine sulfate (0.04 mg/kg) intramuscularly (IM) and then anesthetized with an anesthetic combination of ketamine 10% (90mg/kg) and xylazine 2% (10mg/kg) IM after 10 min from the pre-anesthesia [23]. The back of the anesthetized animals was antisepsis with 1% polyvinyl pyrrolidone iodine and shaved with hair removal cream; then, a formerly heated solid aluminum bar (10mm in diameter) in boiling water (100*°*C) was kept in close contact for about 15 s, with the animal’s skin on the dorsal proximal area.

This pressure equals the mass of 51 g of aluminum bar [24]. For group III (42 rats for burned animals treated with BM-MSCs), the isolated BM-MSCs were suspended in an incomplete DMEM with viability greater than 95% and rapidly injected subcutaneously in the burned animals at a dose of 2 × 10^6^ cells/ml [25].

Animals were observed for 14 sequential days post-burning and treatment for determining the cutaneous clinical course of the wound. The wound retraction was assessed by a caliper in the tested time intervals. Wound contraction was expressed as the reduction in the percentage of the original wound size through using the following formula.

The contracture rate = wound size in the specific day/wound size in the original state × 100% [26].

At the end of each tested time intervals (1 h, 2, 6, 12 and 24 h, 7 and 14 days), animals underwent anesthesia via a mixture of alcohol, chloroform, and ether (ACE) for sacrification; then, skin samples were collected, which were immediately divided into two parts, in which parts of 1 mm^3^ thick were preserved in 10% neutral buffered formalin for 24 h and then transferred into 70% ethyl alc. for the histopathological and immunohistochemical studies, while others were immediately snap-frozen in liquid nitrogen and then preserved under −80^o^C for determining the expression rate of variable wound healing mediators.

### Histopathological study

The previously fixed skin specimens were dehydrated, embedded in paraffin wax, and sectioned at 5 μm, for hematoxylin and eosin staining (H&E) [27].

### Immunohistochemical study

Skin tissue sections from the microarray were cut at a thickness of 4 μm followed by deparaffinization by using xylene, rehydration via descending graded alcohols, followed by antigen retrieval in BT-Link system from DAKO; the activity of the endogenous peroxidase was blocked via using hydrogen peroxide 3%. Primary antibodies were used polyclonal anti-ADAMs10/MADM antibody IgG1, Catalog#YPA2221, Lot: Y15p (Biospes at a dilution rate of 1:50).

The antibody was diluted in phosphate-buffered saline. Then, it was incubated with Power-stain One HRP Polymer (Genemed, USA) for 15 min at room temperature and washed 4 times in buffer. Then, 1 drop of Liquid Fast Red Chromogen was added to 1 ml of Naphthol Phosphate substrate with good mixing. The solution was applied to tissue sections and incubated for 5–10 min. Then, tissue sections were washed with distilled water then counterstained with hematoxylin. After that, the dehydration step was done by using ascending grades of alcohol and xylene. The final step in processing was mounting the slides with a mounting medium and covering with a cover slide. Images of skin tissue sections were analyzed by a freeware version of ImageJ 1.51d (http://rsb.info.nih.gov/ij) [28].

#### RNA isolation and purification for PCR

Extraction of the total RNA was done via the RNA easy Extraction Kit (Qiagen) as follows: 30 mg of frozen skin tissue was thoroughly grist with a mortar and pestle after addition of liquid nitrogen; then, lysis was done with 300 μl of the lysis buffer supplemented with dithiothreitol in a microcentrifuge tube with vortex; then, 600 μl of diluted proteinase K with vortex sec was added and incubated at 15–25°C for 10 min, followed by centrifugation for 5 min at ≥12000 rpm. Precipitation was done via the addition of 450 μl of absolute ethanol to the resultant supernatant in a RNase-free microcentrifuge tube and mix and then transfer up to 700 μl of the lysate to the RNA purification column and centrifuge for 1 min at ≥12000 rpm. Discard the flow-through solution and place the purification column back into the collection tube; repeat this step till all of the lysate has been transferred into the column. Wash three times with the washing buffers supplemented with ethanol with centrifugation for 1 min at ≥ 12000 rpm in each time. For RNA elution, add 100 μl of water nuclease-free to the center of the RNA purification column membrane and centrifuge for 1 min at ≥ 12000 rpm then discard the purification column, and the purified RNA was obtained and preserved at −80 until use.

#### RNA extraction and purification for RT-qPCR

The nucleic acid extraction kit (NucleoSpin®) was used for RNA extraction following the manufacturer’s protocol. For cell lysis, 350 μl of the lysis buffer and 3.5 μl of β-mercaptoethanol were added to the cell pellet and vortex thoroughly, then filtrate the lysate via the filter with centrifugation for 1 min at 11000 rpm, then discard the filter, add 350 μl of 70% ethanol, and mix for adjustment of RNA binding conditions. The lysate was loaded to the column and centrifuged for 30 s at 11000 rpm for binding of the RNA and then the silica membrane was washed twice with the washing buffers and centrifuge for 30 s at 11000 rpm; for full membrane dryness, the RNA column was centrifuged for 2 min at 11000 rpm. For RNA elution, the RNA column was placed into a nuclease-free collection tube then RNA was eluted in 60 μl DNase-free H_2_O and centrifuged for 1 min at 11000 rpm. The purified RNA samples were kept at −80 °C for further use.

#### Quantitative assay of the mRNA rates of TGF-β and the pro-inflammatory cytokines (IL-6 and TNF-α) via PCR

Extraction of the total RNA was done via the RNA easy Extraction Kit (Qiagen) according to the manufacturer’s guidelines. Both yield and purity were assessed at 260 and 280 nm respectively using a Nanodrop ND-2000 spectrophotometer (Thermo Electron). A total amount of 1 μg RNA was used for cDNA synthesis by Viva 2-steps RT-PCR Kit according to the manufacturer’s instructions.

Quantitative PCR using Thermo Scientific Verso 1-step RT-PCR Ready-Mix kit (Applied Biosystems, Foster City, CA, USA) was conducted to analyze the levels of the mRNA of the target genes which quantified relatively in relation to the expression rate of β-actin. The primers of amplification include IL-6 F: 5′-GCCT TCTTGGGACTGATG-3, R: 5′-TGGTCTGTTGTGGGTGGT-3′, TNF-α F: 5′-GCTGAGGTTGGACGGATAAA-3′, R: 5′-AAAATCCTGCCCTGTCACAC -3′, and TGF-β F: 5′-TGGCGTTACC TTGGTAACC- 3′, R: 5′- GGTGTTGAGCCCTTTCCAG- 3′, and B-actin, F: 5-d TCCCTGAAGTACCCCATGGAG-3′, R: 5′-d TTGGCCTTGGGGTTCAGGGGG-3.

#### Quantitative assay of the mRNA levels of HSP-90α, MMP-9, and miR-21 genes via RT-qPCR

The purity (A260/A280 ratio) and the concentration of RNA were detected using spectrophotometry (dual-wavelength Beckman, spectrophotometer, USA). cDNA synthesis was done via Vivantis, ViPrimePLUS One Step Taq RT-qPCR Green Master Mix I with ROX (SYBR Green Dye) (cat no #QLMM14-R) kit according to the protocol of the manufacturer.

The prepared reaction mix samples were applied in real-time PCR (Step One Applied Biosystem, Foster City, USA) to analyze the mRNA expression rate of the specific genes. The primers used are β-actin F: TGACAGGATGCAGAAGGAGA, R: TAGAGCCACCAATCCACACA; HSP-90 F: TGTTGGGACCAGCAACTCAA, R: TTTGAGGCTCAGTGGTAGCC; MMP-9 F: GGCAGCTTCAACAACCATCA, R: GGATGGACTAGATCGGAGCC; 6UB F: AACGCTTCACGATTTGCGT, R: CTCGCTTCGGCAGCACA; and miR- 21 F: TAGCTTATCAGACTGATGTTGA, R: GAATCGAGCACCAGTTACGC. The RQ of each target gene is quantified through the calculation of the delta-delta Ct (ΔΔCt), and the RQ of each gene was calculated via taking 2^−∆∆Ct^.

### Statistical analysis

The results are expressed as the mean values ± standard error (SE). Continuous variables were analyzed with the one-way analysis of variance (ANOVA), followed by the Duncan post hoc test. A *P* value of <.05 was considered significant statistically. All data analyses were done via IBM SPSS Statistics ver. 22.0 (IBM Co., Armonk, NY, USA).

## Results

### Clinical evaluation

**Fig. 1:**
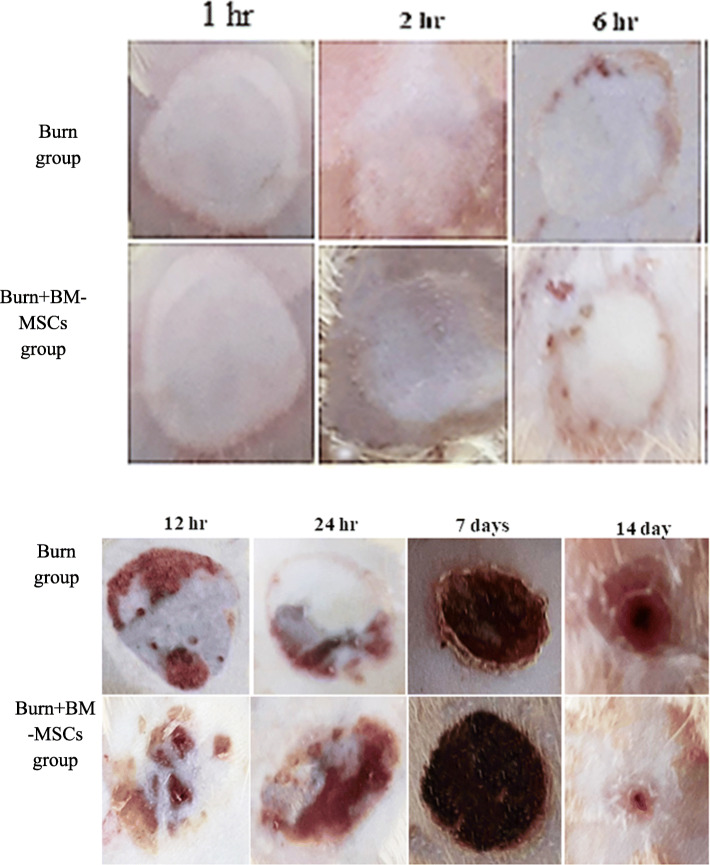
Macroscopic appearance of the burn wound and the treated wound with BM-MSCs

**Fig. 2:**
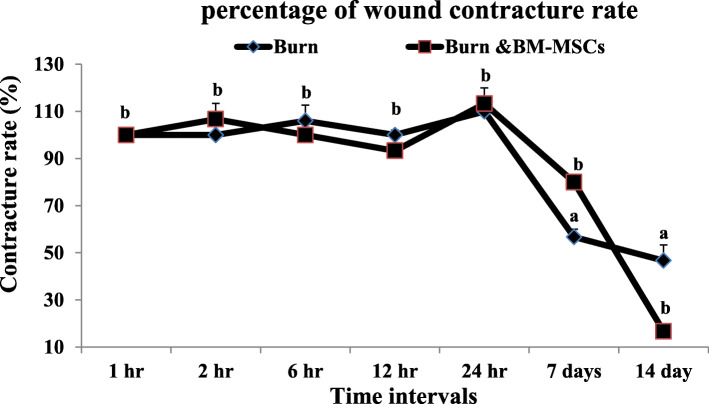
Percentage of the contracture rate of the burn wound and the treated wound with BM-MSCs

The pale circular lesion has been noticed grossly following the application of the previously heated aluminum bar in both treated and non-treated animals; then, a blister was developed at 2 h which becomes pale at 6 h; after that, the ruptured blisters were developed at 12 h and increased at 24 h markedly in the treated animals with BM-MSCs. At 7 days, the lesion was covered with the reddish stiff crust which decreased in size and still attached to the wound till 14 days in a burned animal only while in treated animals the wound assumed pinpoint appearance and the skin nearly appeared normal (Fig. 1). Figure 2 reveals a significant elevation of the percentage of the wound contracture rate at 7 days which decreased markedly at 14 days in the treated animals with BM-MSCs (G3) in comparison to the burned animals (G2).

### Histopathological findings

**Fig. 3:**
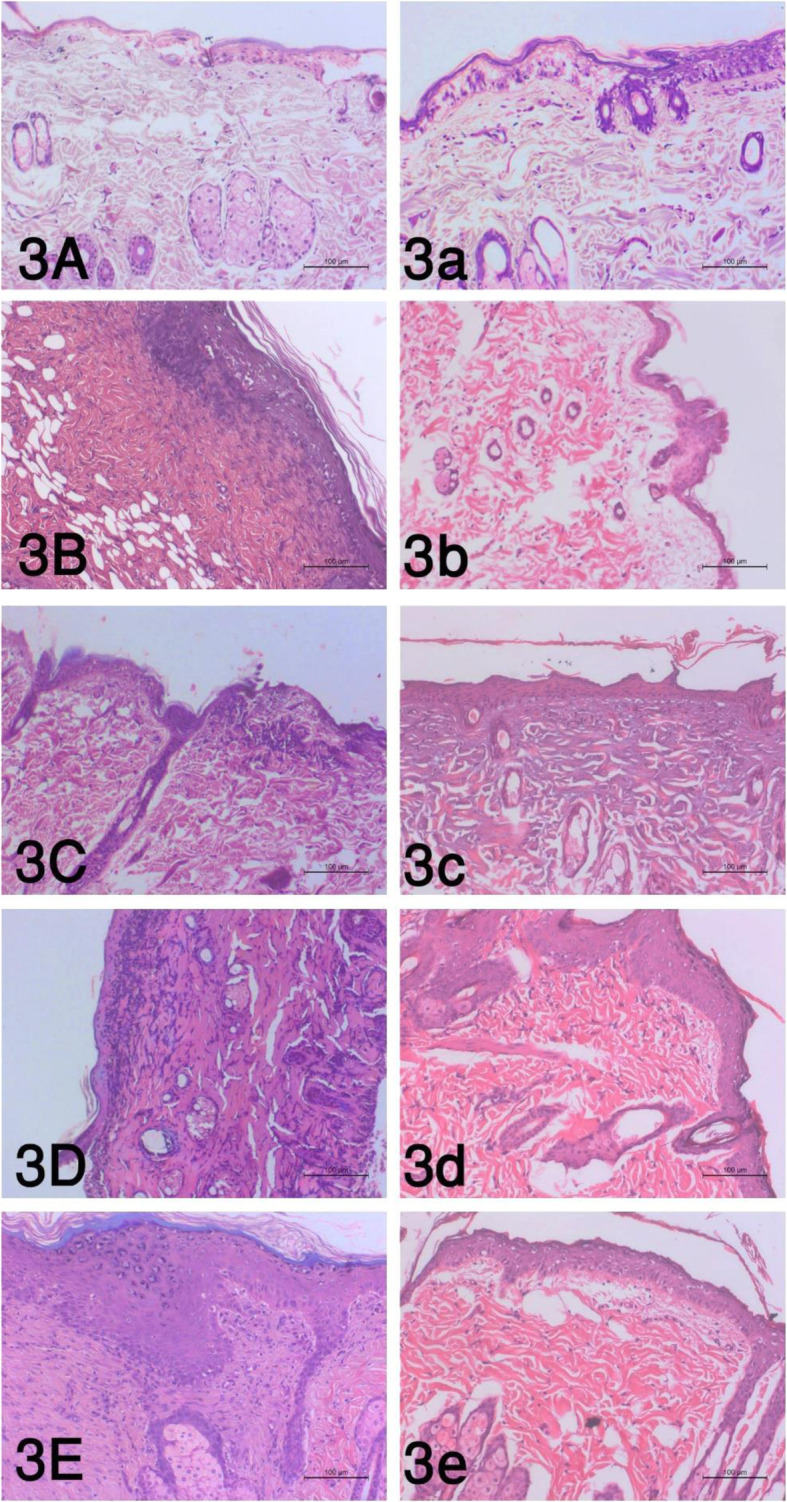
The histopathological alterations of the burn wound at the tested time intervals as follows: **A** 1 h, **B** 6 h, **C** 24 h, **D** 7 days, and **E** 14 days, and the treated wound with BM-MSCs as follows: **a** 1 h, **b** 6 h, **c** 24 h, **d** 7 days, and **e** 14 days (*P* value <.05)

**Fig. 4:**
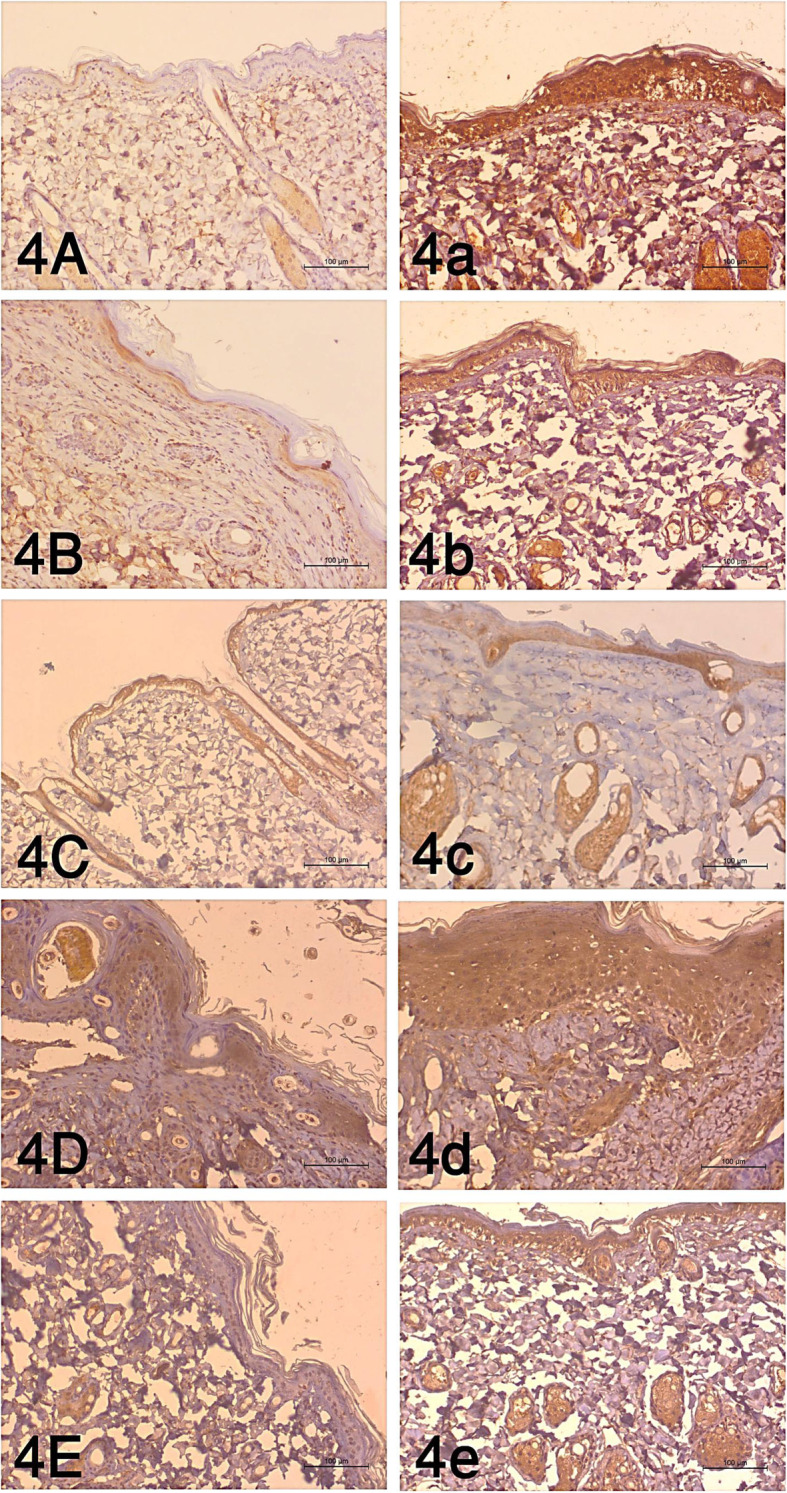
The immunohistochemical examination of the burn wound at the tested time intervals as follows: **A** 1 h, **B** 6 h, **C** 24 h, **D** 7 days and **E** 14 days, and the treated wound with BM-MSCs as follows: **a** 1 h, **b** 6 h, **c** 24 h, **d** 7 days, and **e** 14 days (*P* value <.05)

Histopathological examination of skin sections of the control group revealed the normal histological structure of the skin that includes the epidermis which is composed of stratified squamous epithelium and dermis. Skin sections of albino rats in both burned animals and treated animals with BM-MSCs at 1 h time showed severe degenerative changes and necrosis of the squamous epithelium lining associated with the absence of the epidermis in certain burn areas that indicate deep second-degree burn (Fig. 3A and 1a); also, damaged hair follicles could be found with loss of the dermal architecture leaving empty spaces (edema) associated with the presence of coagulative acidophilic fused collagen fibers at 6 and 24 h post-burning (Fig. 3B, C),

In contrast, quite an improvement of the epidermal and dermal layers could be detected in the treated animals at 6 and 24 h (Fig. 3b and 4c). At 7 days post-burning, the epidermal layer showed acidophilic scab formation; furthermore, granulation tissue formed in the dermal layer could be found (Fig. 3D). Epidermal growth with immature differentiation and dermal granulation tissue formation have been evident at 14-day post-burning (Fig. 3E) and 7-day post-BM-MSC injection (Fig. 3d), while at 14-day post-injection, the epidermal layer was regularly arranged with thin stratum corneum and the underlying granulation tissue of the dermis showed few inflammatory cells and edema (Fig. 3e).

### Immunohistochemical findings

The area percentages of ADAMs10 immunohistochemical expression in the burn group (G2) were 16.7, 14.6, 16.3, 24.95, and 28.9 in 1 h, 6 h, 24 h, 7 days, and 14 days respectively. Means of the area percentages of the treated group with BM-MSCs (G3) were 28.9, 27.1, 19.6, 31.6, and 9.1 in 1 h, 6 h, 24 h, 7 days, and 14 days respectively with the highest percentages at 7 days. Statistical analysis of all possible pairwise comparisons revealed a significant increase at 1 h time and 6 h and a significant decrease at 14 days as shown in Table 1 and Fig. 4 (*P* value <0.05).

**Table 1:** Immunohistochemical expression of ADAMs10 area percentages (%) in the burn wound and the treated wound with BM-MSCs

Time intervals	Burn	Burn and BM-MSCs
**1 h**	16.7±1.8^a^	28.9±3.2^b^
**6 h**	14.6±1.7^a^	27.1± 0.7^b^
**24 h**	16.3±4.9^b^	19.6±1.3^b^
**7 days**	24.95±4.1^b^	31.6±4.9^b^
**14 days**	28.9±3.2^a^	19.1 ± 1.3^b^
***P-*value**	***P*< 0.05**	

^a,b^Means which have the similar superscript symbol(s) are not significantly different. Data are expressed as mean ± SE (*n*=6)

### mRNA expression rate of TGF-β, IL-6, and TNF-α

**Fig. 5:**
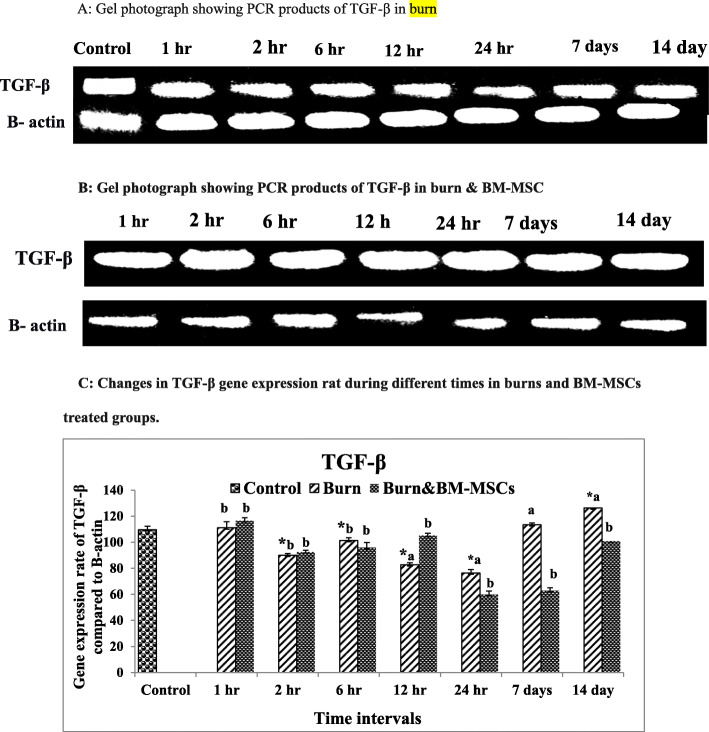
mRNA expression rate of TGF-β in burn wound and the treated wound with BM-MSCs (*P* value <.05). **a** Gel photograph showing PCR products of TGF-β in the burn. **b** Gel photograph showing PCR products of TGF-β in the burn and BM-MSC. **c** Densitometric analysis of PCR products of TGF-β

The burned animals (G2) showed significant upregulation in TGF-β expression rate at 14 days, while the significant downregulation was reported at 2, 6, 12, and 24 h in comparison to the control group (G1). Contrary, S/C injection of BM-MSCs causes significant upregulation at 12 h with significant downregulation at 24 h and 7 and 14 days (Fig. 5) (*P* value <0.05).

**Fig. 6:**
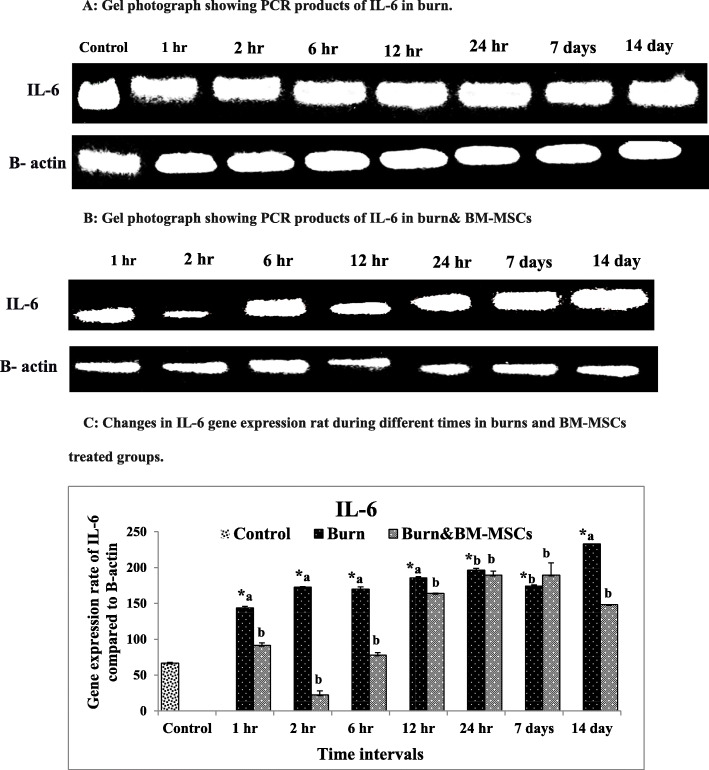
mRNA expression rate of IL-6 pro-inflammatory cytokine in burn wound and the treated wound with BM-MSCs (*P* value <.05). **a** Gel photograph showing PCR products of IL-6 in the burn. **b** Gel photograph showing PCR products of IL-6 in the burn and BM-MSCs. **c** Densitometric analysis of PCR products of IL-6

Regarding the mRNA expression rate of IL-6, the rate was significantly upregulated in the burn group (G2) at all intervals in comparison to the control group (G1). On the other hand, BM-MSCs resulted in significant downregulation at 1 h, 2, 6 h, and 12 h and 14 days (Fig. 6) (*P* value <0.05).

**Fig. 7:**
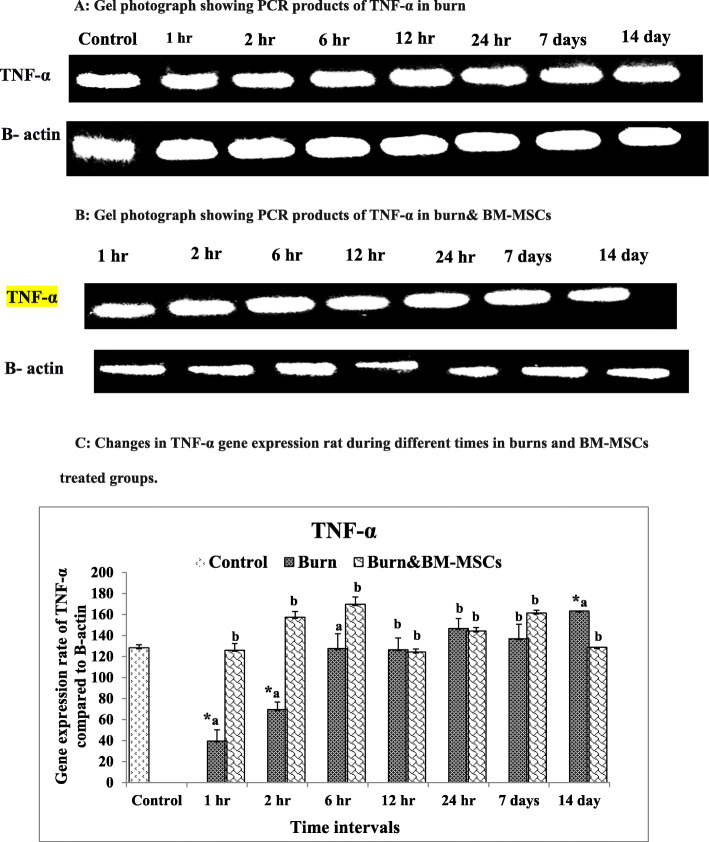
mRNA expression rate of TNF-α pro-inflammatory cytokine in the burn wound and the treated wound with BM-MSCs (*P* value <.05). **a** Gel photograph showing PCR products of TNF-α in the burn. **b** Gel photograph showing PCR products of TNF-α in the burn and BM-MSCs. **c** Densitometric analysis of PCR products of TNF-α

For the mRNA TNF-α expression rate, the burned animals exhibited significant downregulation at 1 h and 2 h time; however, the significant upregulation was reported at14 days in comparison to the control group (G1). Following BM-MSC injection, the remarkable upregulation was recorded at 1 h, 2, and 6 h time with significant downregulation at 14 days (Fig. 7) (*P* value <0.05).

### mRNA expression rate of HSP-90α, MMP-9, and miR-21 genes

**Fig. 8:**
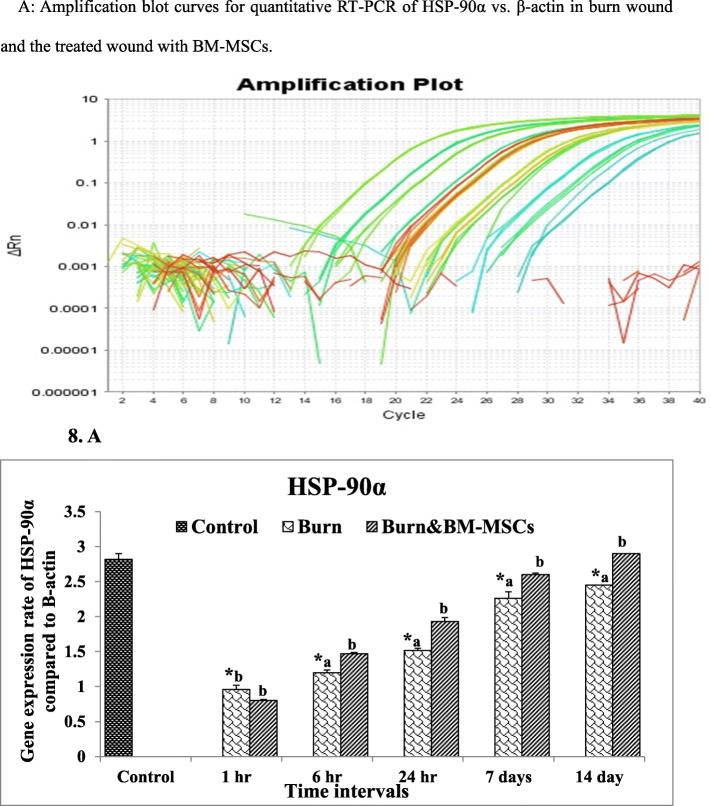
The mRNA expression rate of HSP-90α gene in the burn wound and the treated wound with BM-MSCs (*P* value <.05). **a** Amplification blot curves for quantitative RT-PCR of HSP-90α vs. β-actin in the burn wound and the treated wound with BM-MSCs

Figure 8 illustrates that the expression rate of HSP-90α was significantly downregulated in all-time intervals of burn healing in comparison to the control group (G1). After S/C injection of BM-MSCs (G3), the HSP-90α expression rate was significantly upregulated at 6 and 24 h and 7 and 14 days (*P* value <0.05).

Concerning the MMP-9 expression rate, it was upregulated significantly at all-time intervals of wound healing in comparison to the control group (G1).

**Fig. 9:**
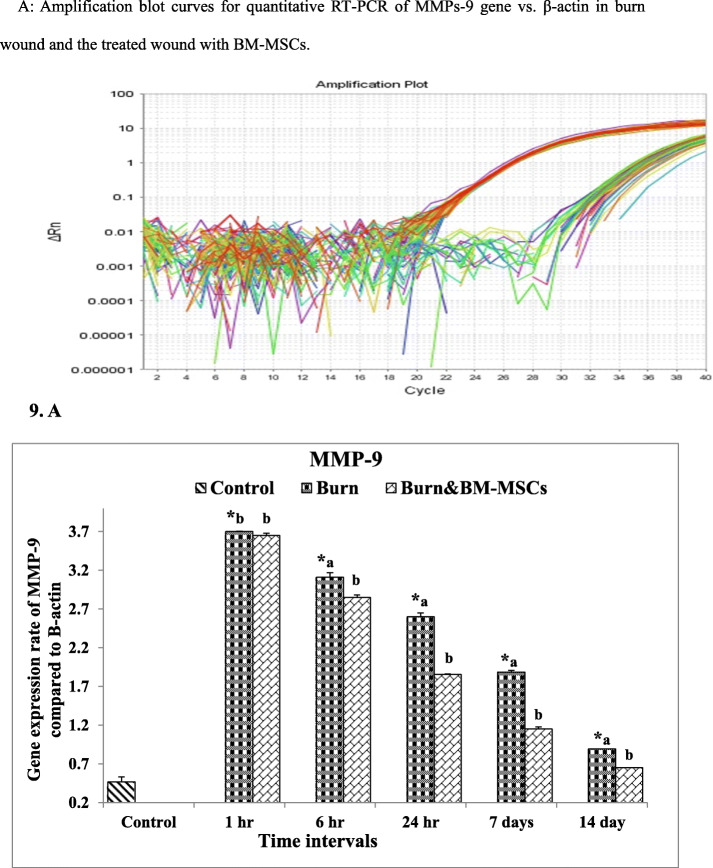
The mRNA expression rate of MMP-9 gene in the burn wound and the treated wound with BM-MSCs (*P* value <.05). **a** Amplification blot curves for quantitative RT-PCR of MMPs-9 gene vs. β-actin in the burn wound and the treated wound with BM-MSCs

**Fig. 10:**
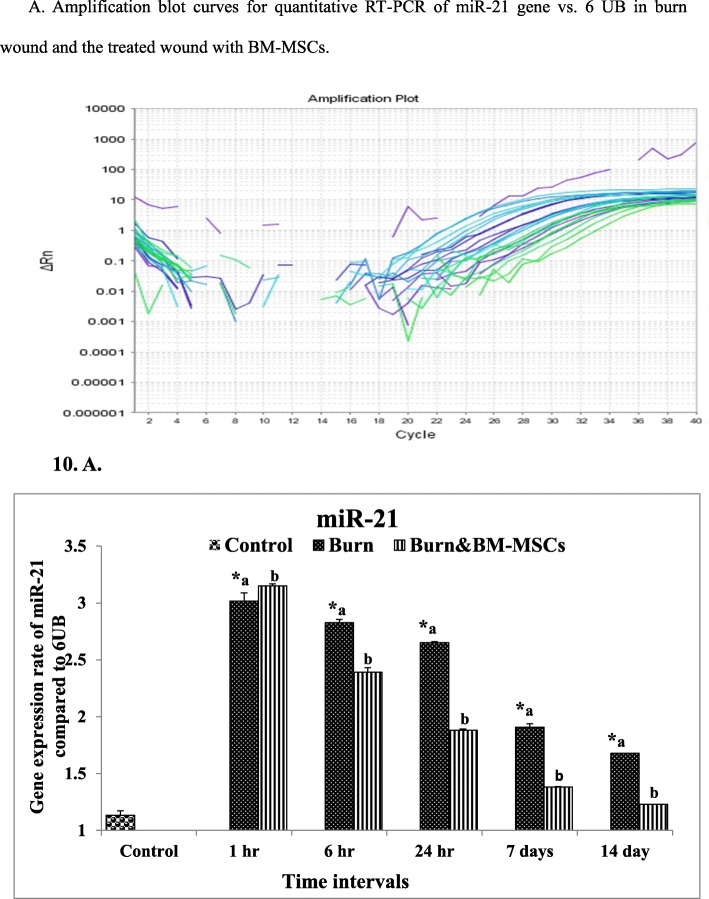
The mRNA expression rate of miR-21 gene in the burn wound and the treated wound with BM-MSCs (*P* value <.05). **a** Amplification blot curves for quantitative RT-PCR of miR-21 gene vs. 6 UB in the burn wound and the treated wound with BM-MSCs

On the other hand, treatment with BM-MSCs causes significant downregulation at 6 and 24 h and 7 and 14 days (Fig. 9) (*P* value <0.05). In comparison, the expression rate of miR-21 between burned (G2) and control (G1) groups is shown in Fig. 10 and the results revealed significant upregulation at all-time intervals; in contrast, the rate was upregulated markedly at 1 h time and significantly downregulated at 6 and 24 h and 7 and 14 days following the treatment with BM-MSCs (*P* value <0.05).

## Discussion

Annually, more than 250,0000 deaths happen because of burns; in addition, severe economic, physical, and psychological losses resulted from the disabilities and disfigurements which resulted from the non-fatal ones [29]. Hence, they are considered one of the most serious types of thermal trauma, and the body reacts with this health problem through different mechanisms including cellular protection, inflammatory response, immune suppression, and hypermetabolic dysfunction [30].

For restoring the protective function of the skin after the cutaneous injury, several well-orchestrated cooperative dynamic processes have been occurred and represented in four successive phases including hemostasis, inflammation, proliferation, and remodeling [31]. The indicators of wound healing as the acceleration of re-epithelization and thickness of the regenerated epidermis have been recorded post-treatment with BM-MSCs [32]. After burn injury, BM-MSCs are engrafted to the injured area for regeneration through transdifferentiate and interaction with the epithelial cells [33].

Furthermore, the inherent MSCs drafted to the wound area and become activated by the inflammatory medium and in close interaction with the immune system in a process called licensing [34] and begin to produce the growth factors and cytokines resulting in alteration of the composition of the local cytokines which are beneficial for wound healing and tissue regeneration processes [35]. In the current study, we reported a rapid response in both pro-inflammatory cytokines (TNF-α and IL-6) during the short periods (1, 2, and 6 h) in the inflammatory stage, during which BM-MSCs increased significantly and ameliorated these changes.

The wound healing process enhanced via inhibiting the inflammatory process, increasing angiogenesis, stimulating the migration of fibroblasts, and collagen production through paracrine mechanisms [35]. Furthermore, their paracrine factors cause downregulation of the nucleic acid, protein metabolism, and apoptotic genes, with upregulation of the homeostatic and anti-apoptotic genes [36].

S/C injection is a convenient way for introducing BM-MSCs into the injured area [37], which proves to achieve the indicators of the healing process as elevating the angiogenesis and the density of the capillaries that could be observed by the naked eye and proved with the CD31, Ang-1/2, and VEGF expression [38].

The immunomodulation property of MSCs enables them to attenuate the inflammatory response directly through inhibiting the production of the pro-inflammatory cytokines as TNF-α and IFN γ with the simultaneous elevation of the secretion of the anti-inflammatory cytokine such as IL-10 and IL-4 [39], besides their inhibitory effect on neutrophil infiltration and IL-6 [40], and the successful wound healing is achieved through resolution of inflammation [41]. Thus, the rate of IL-6 and TNF-α was markedly downregulated after S/C injection of BM-MSCs in the burned animals. Upregulation of TGF-β was reported via Caliari-Oliveira et al. [42] and Gilbert et al. [43] in full-thickness burn injury; on the other hand, a marked reduction in TGF-β expression was reported in the current study of a deep second-degree burn.

The family of MMPs involves the membrane-bound MMPs, the classical MMPs, and the ADAMs [44]. The expression rate and the activity of gelatinase MMPs (MMP9 and MMP2) were significantly upregulated through TGF-β, in which its expression rate markedly downregulated with inhibition of miR-21 [45]; other results reported that the miR-21 was in-dispensable for the migration of the TGF-β-driven keratinocyte in vitro and could enhance the process of re-epithelialization during healing [46].

ADAMS 10 are responsible for extracellular matrix degradation, patterns of the cell signaling, and localized shedding of different proteins of the cell surface [47]. For example, it is considered the main E-cadherin sheddase [48].

Different mechanisms such as transcriptional and translational control are responsible for ADAM10 activity [49], in which Adam10 and Adam17 are responsive to TGF-β transcriptional regulation directly [50], TGF-β has mediated the ADAMS upregulation because of their inhibitory effect on the repressors as the ski-related novel protein N in a Smad2/3-dependent manner [51], which may be the reason for decreasing the area percentages of ADAMs10 immunohistochemical expression at 14 days post-treatment with BM-MSCs.

The MSCs or MSC-conditioned medium hasten the wound closure due to their abilities to stimulate the dermal fibroblasts which produce a huge amount of collagen type I and change the gene expression [52], leading to the promotion of the wound healing [53]; moreover, MSCs have anti-scarring property via secretion of VEGF and HGF and preserving the equilibrium between TGF-β1 and TGF-β3 under the influence of their paracrine signaling [54], which was identified in the current study.

Li et al. [55] reported that growth factors are not the only factors responsible for wound closure, but also Hsp90α has a significant role in this process via promoting the cell survival and motility [56]. Moreover, it was responsible for the migration of human epidermal and dermal fibroblasts [57]. Regarding the epithelization process, BM-MSCs enhanced this process by promoting the proliferation of resident epidermal cells in the presence of EGF or may be differentiated into epidermal cells [58, 59].

There was another clinical application for treating wound comparing with our study including stromal vascular fraction cells (SVFs) representing the main source for adipose-derived stem cells (ASCs) that may be incorporated in different scaffolds [60] and utilized for therapy of thermal wounds, scars, and injuries of cartilage and bone as a way of regenerative surgery [61]. Moreover, the process of wound healing has been enhanced following the use of autologous growth factors derived from the blood platelets [62] such as platelet-rich plasma (PRP) [63]. They have the capability to stimulate cellular proliferation and differentiation and neoangiogenesis [61].

In an alternative study, re-epithelization of soft and hard tissue wounds has been achieved following dressing with PRP and hyaluronic acid (HA) [64]. Moreover, a safe and fast wound healing closure of Hidradenitis suppurativa (HS) without any complications, infection, and recurrence has been achieved through the surgical excision and closure using PRP gel and Hyalomatrix PA (HPA) [65]. Scioli et al. [66] observed that the chondro-/osteogenic differentiation of ASCs has been improved in the 3D collagen scaffold culture supplemented with insulin and platelet-derived growth factors.

On the level of clinical application of PRP in treating hair problems, using a combination of PRP and micrografts containing human follicle MSCs (HF-MSCs) was reported to be a safe and effective therapy for treating patients with androgenetic alopecia (AGA) [67]. Positive effects have been detected in the hair growth following exposure to MSCs and platelet-derived growth; they produced signaling affecting the hair growth through cellular proliferation to expand the anagen phase, stimulating the development of the hair follicle (β-catenin), stimulating the cell growth, and inhibiting the apoptotic cues (Bcl-2 release and Akt activation) [67].

Application of enhanced stromal vascular fraction (e-SVF) could enhance tissue healing and maintenance of fat graft volume in posttraumatic lower extremity ulcers [64] and breast reconstruction [68], in which Celution and Fat stem proved to be the best two automatic systems to obtain SVF with enhancing maintenance of the fat volume and preventing the reabsorption [69]. Furthermore, the more favorable results in terms of clinical outcome and yield of SVFs have been obtained from using the Supercharged-modified nano-fat in treating scars [70]. In breast reconstruction surgery, using the engineered fat graft enhanced with adipose-derived stromal vascular fraction cells (EF-e-A) was proved to be a safe and effective therapy [71].

## Conclusion

Subcutaneous injection of BM-MSCs leads to great improvements in the healing process of the deep second-degree burn through downregulating the expression rate of the IL-6, TNF-α, TGF-β, MMP-9, and miR-21; marked upregulation of HSP-90α particularly at late stages of wound healing; restoring the normal skin architectures; and reducing the scar tissue formation. Therefore, the skin appeared approximately normal. BM-MSCs enhance tissue healing response, involving cellular inflammatory, angiogenesis, and molecular and matrix deposition.

## Data Availability

The data supporting the conclusions of this article are included within the article.
